# Effects of Age on Compounds, Metabolites and Meat Quality in Beijing-You Chicken Breast Meat

**DOI:** 10.3390/ani13213419

**Published:** 2023-11-04

**Authors:** Xia Chen, Jing Cao, Cheng Chang, Ailian Geng, Haihong Wang, Qin Chu, Zhixun Yan, Xiaoyue Zhang, Yao Zhang, Huagui Liu, Jian Zhang

**Affiliations:** Institute of Animal Husbandry and Veterinary Medicine, Beijing Academy of Agriculture and Forestry Sciences, Beijing 100097, China; chenxia_91@163.com (X.C.); caojing2046555@163.com (J.C.); changeng02@163.com (C.C.); ailiangengcau@126.com (A.G.); haioulantian@126.com (H.W.); chuqinsd@163.com (Q.C.); yanzhixun2008@sina.com (Z.Y.); 13240150468@163.com (X.Z.); duguyimeng1@126.com (Y.Z.)

**Keywords:** Beijing-You chicken, breast meat, age, meat quality, free amino acid, metabolites, flavor

## Abstract

**Simple Summary:**

Age plays a critical role in meat quality and flavor formation. A clear understanding of flavor changes during development is helpful to regulate meat quality as well as flavor in production. Various compounds and metabolites are the sources of flavor precursors, which contribute most to flavor development. Beijing-You chicken, famous for its special appearance and excellent flavor, is an excellent model to study the changes in meat quality and flavor against age. Therefore, the compounds, metabolites, and meat quality in Beijing-You chicken breast meat at the ages of 90, 120, and 150 days were characterized to trace their changes against age. It was found that the meat quality and metabolites at 90 days of age were significantly different from those at the ages of 120 and 150 days, revealing that attention should be focused on the period of 90 to 120 days to study the flavor formation mechanism in Beijing-You chicken breast meat. This study provides an initial insight into the changes in compounds, metabolites, and meat quality in chicken meat around market ages.

**Abstract:**

The physical properties, free amino acids, and metabolites of Beijing-You chicken (BYC) breast meat aged 90, 120, and 150 days were analyzed to investigate the flavor changes with age. The shear force and intramuscular fat increased from 90 to 120 days significantly. The contents of total free amino acids and essential amino acids decreased from 90 to 120 days significantly. No significant differences were detected between 120 and 150 days. The contents of sweet amino acids, bitter amino acids, and umami amino acids showed no significant differences between different ages. In addition, GC-MS and LC-MS were integrated for metabolite detection in breast meat. A total of 128, 142, and 88 differential metabolites were identified in the comparison groups of 120 d vs. 90 d, 150 d vs. 90 d, and 150 d vs. 120 d. Amino acids and lipids were the main differential metabolites. The pathway analysis showed that arginine biosynthesis, histidine metabolism, purine metabolism, and cysteine and methionine metabolism were the main pathways involved in flavor formation during BYC development. It was also found that the metabolites associated with flavor, such as methionine, cysteine, glucose, anserine, arachidonic acid, and glycerol 1-phosphate, were significantly affected by age.

## 1. Introduction

With the improvement of living standards, the demand for high-quality poultry products is increasing. The texture, flavor, and nutrition of meat are important indexes for meat quality evaluation. The physical properties (color, tenderness, and juiciness) of meat influence the texture of meat and directly affect consumers’ desire [1,2,3]. The composition of free amino acids, peptides, fatty acids, organic acids, aldehyde, vitamins, etc., determines the flavor and nutrition of meat [4]. For instance, amino acids are the raw materials of protein synthesis and have important roles in food flavor. In addition, free amino acids also play an important role in food flavor. Glutamic acid and aspartic acid are important contributors to umami [1]. Serine, proline, glycine, threonine, and alanine have a sweet taste, while arginine, valine, tyrosine, tryptophan, phenylalanine, leucine, and isoleucine have a bitter taste [5]. Lipids in meat are the source of essential fatty acids [6]. Moreover, lipids can be degraded to volatile compounds during cooking through the Maillard reaction and Strecker degradation [7]. Therefore, lipids also play a crucial role in flavor development. 

Meat flavor relies heavily on the flavor precursors that derive from the metabolites in meat. Therefore, analysis of metabolites in meat could provide us some indicative insights into the flavor formation mechanism. Metabolomics is popular for efficient analysis of small molecules or metabolites in a cell and tissue. Gas chromatography–mass spectrometer (GC-MS) and liquid chromatography–mass spectrometry (LC-MS) are two powerful analytical platforms for metabolomics [8]. LC-MS shows advantages in semi-polar metabolites’ and macromolecular components’ investigation [9]. Volatile and thermostable compounds contributing to the odor of meat can be effectively detected using GC-MS [9]. In recent years, metabolomics has been applied in the study of meat quality and flavor. Setyabrata et al. (2021) identified the flavor-related chemical compounds of dry-aged beef using LC-MS and found that glutathione metabolism could contribute to dry-aged flavor generation [10]. Yu et al. (2021) integrated GC-MS and LC-MS to analyze the effect of cooking methods on water-soluble compounds and flavor compounds in Chinese Piao chicken and found that 3-methylbutyraldehyde was the main volatile flavor compound in roasted chicken [11]. The differential metabolites and lipids in skeletal muscle were identified to explore the differences in flavor precursors between Laiwu and Yorkshire pigs [12].

The meat quality and flavor are affected by various factors, such as breed, gender, age, nutrition, and environment. Age is generally considered a non-genetic factor that plays vital roles in meat quality, flavor, and nutrition formation [13]. Beijing-You chicken (BYC) was listed as one of the most important chicken breeds in 2011 and joined the “Agro-product Geographical Indications” in 2020. BYC is popular among consumers for its unique appearance, excellent meat quality, and special flavor [14], which make it an excellent model to study the influence of age on meat quality and flavor. Generally, the marketable age of BYC is 120 d. It is still unclear how the meat quality and flavor vary around the market age. Therefore, the effects of age on compounds, metabolites, and meat quality were studied from breast meat of BYC at 90, 120, and 150 days of age. 

## 2. Materials and Methods

### 2.1. Sample Collection

A total of 90 one-day-old female BYC were provided by the Institute of Animal Husbandry and Veterinary Medicine, Beijing Academy of Agriculture and Forestry Science. All birds were housed in an environment-controlled room with food and water available ad libitum. At the ages of 90, 120, and 150 d, 15 birds were randomly selected for each group (Table S1). After 12 h of overnight fasting, all the birds were weighed and bled to death. Breast meat (pectoralis major) was collected. The left breast meat was flash-frozen in ambient liquid nitrogen and then stored at −80 °C, which was used for metabolomics analysis. The right breast meat was prepared for physical properties’ and free amino acid analysis.

### 2.2. Physical Properties 

#### 2.2.1. Water-Holding Capacity

Drip loss and cooking loss were measured for meat’s water-holding capacity (WHC). Meat samples were trimmed and visible fat was removed. The meat sample for drip loss evaluation was first weighed (W1) and stored in an airtight box at 4 °C. After 24 h, the sample was reweighed (W2). The difference between W1 and W2 is defined as the drip loss. 

For cooking loss, the meat sample was stored at 4 °C for 24 h and weighed (W3). Then, the sample was put in a vacuum-sealed bag for storage individually. Afterward, it was cooked in a water bath at 85 °C till the internal temperature reached 80 °C. When the cooked sample cooled to room temperature, it was dried and reweighed (W4). The drip loss and cooking loss were calculated as below:

$$\mathrm{Drip}\mathrm{loss}\left(\%\right)=\frac{W1-W2}{W1}\times 100$$

$$\mathrm{Cooking}\mathrm{loss}\left(\%\right)=\frac{W3-W4}{W3}\times 100$$

#### 2.2.2. Shear Force

The meat after cooking loss characterization was cut into strips parallel to the direction of muscle fiber for shear force measurement. The meat’s shear force was measured using TMS-PRO (FTC Co., Sterling, VA, USA) at a speed of 200 mm/min. Each sample was measured at least three times and the shear force was determined as the average value of all the measurements. 

#### 2.2.3. Dry Matter 

The weight of fresh meat sample without visible fat was first recorded (W5). Then, it was freeze-dried for 60 h in a freeze-dryer (Millrock Technology, Kingston, NY, USA) to dry the meat. Finally, the dried meat was reweighed (W6). The relative difference between W5 and W6 was defined as the dry matter content:

$$\mathrm{Dry}\mathrm{matter}\left(\%\right)=\frac{W5-W6}{W5}\times 100$$

#### 2.2.4. Intramuscular Fat

Intramuscular fat (IMF) was extracted using Soxhlet extraction. First, a clean and dry reservoir bottle was prepared and weighed (W7). Then, a 5 g meat sample (dried with a freeze-dryer) was mixed with 100 mL petroleum ether. Afterward, the reservoir bottle was dried and reweighed (W8). Finally, the IMF content was calculated:

$$IMF\left(\%\right)=\frac{W8-W7}{5}\times 100$$

### 2.3. Free Amino Acids

A mixture of 1 g degreased meat lyophilized powder and 25 mL hydrochloric acid (0.1 mol/L) was blended for 15 min to fully mix. Then, it was centrifuged for another 15 min at 4000 rpm. The supernatant was collected. Then, 2 mL sulfosalicylic acid (0.2 mol/L) was added to 2 mL supernatant and centrifuged for 15 min at 3500 rpm. The mixture was further filtered through a 0.45 μm membrane. Finally, the mixture was analyzed with an amino acid analyzer (L-8900, Hitachi Ltd., Tokyo, Japan).

### 2.4. Liquid Chromatography–Mass Spectrometry 

The detailed procedures of LC-MS profiling have been described in Chen et al. (2023) [15]. The metabolites were extracted from 1 mL mixture of methanol, acetonitrile, and water with a volume ratio of 2:2:1 and stored as powder at −80 °C. To demonstrate the equipment stability as well as data reliability in LC-MS analysis, a quality control (QC) sample, where all experimental analytes were equally pooled, was also prepared. The LC-MS profiling was conducted through Agilent 1290 Infinity LC coupled with a mass spectrometer Triple-TOF 5600 from AB Sciex. The extracted metabolite powders were dissolved in 100 μL acetonitrile aqueous solution, where acetonitrile and water were mixed with an equal volume. All the extracted powders were analyzed via electrospray ionization with both positive and negative modes adopted. 

### 2.5. Gas Chromatography–Mass Spectroscopy 

For GC-MS profiling, a very detailed procedure can be found in Chen et al. (2023) [15]. The metabolites were first extracted using methanol and ribitol and then analyzed on a system made up of an Agilent 6890A gas chromatograph and 5973C mass spectrometer. Full scan mode was used during MS analysis with a detectable spectrum range from 50 to 550 (*m*/*z*).

### 2.6. Statistical Analysis 

Prior to significance testing, normality tests were first performed on the data. Different significance testing methods were adopted based on the normality test result. ANOVA was used if the data had a normal distribution; otherwise, a Wilcoxon test was used. The XCMS package was used for feature detection, peak alignment, and retention-time correction in the measurement data. Metabolites were identified by searching the standard MS database with the criterion of precise mass <25 ppm. Note that data with missing values over 50 percent were considered invalid and excluded. Afterward, principal component analysis (PCA) and orthogonal partial least-squares discriminant analysis (OPLS-DA) were applied to the data in the ropls package in R (version 4.1.2). The heatmap and volcano diagram in this study were plotted in R. The percentage stacking bar plot was drawn in Excel (version 2019).

## 3. Results

### 3.1. Physical Properties’ Analysis

To study the influence of age on meat texture, we evaluated the physical properties of breast meat at the ages of 90, 120, and 150 days (Table 1). Compared to 90 and 150 d, breast meat at 120 d had the lowest drip loss and cooking loss (*p* < 0.05). The shear force and IMF content increased significantly from 90 to 120 d (*p* < 0.05) and had no significant difference at 120 and 150 d (*p* > 0.05). 

### 3.2. Amino Acid Analysis

To investigate the changes in free amino acids at different ages, the contents of free amino acids in breast meat of BYC were quantitatively analyzed with an amino acid analyzer. A total of 15 amino acids were detected in breast meat, among which glutamic acid, threonine, and alanine were the top three most abundant amino acids (Table 2 and Table S2). The content of glutamic acid increased with age, but it did not reach the significant level (*p* > 0.05). The content of threonine had no significant difference between 90, 120, and 150 d. The content of alanine at 150 d was significantly lower than that at 90 and 120 d (*p* < 0.05), yet it did not show a significant difference between 90 and 120 d. Regarding methionine, the content was the highest at 150 d while it showed no significant difference between 90 and 120 d.

In addition, the contents of total free amino acids and essential amino acids at 90 d were significantly higher than that at 120 and 150 d (*p* < 0.05, Table 3). Since free amino acids have important effects on taste, the contents of sweet amino acids, bitter amino acids, and umami amino acids were further analyzed. The contents of umami amino acids and sweet amino acids showed no significant differences among the three ages (*p* > 0.05). As for bitter amino acids, they suffered a significant decrease from 90 to 120 d (*p* < 0.05). 

### 3.3. Metabolomics Analysis

#### 3.3.1. PCA and OPLS-DA Analysis

**Figure 1:**
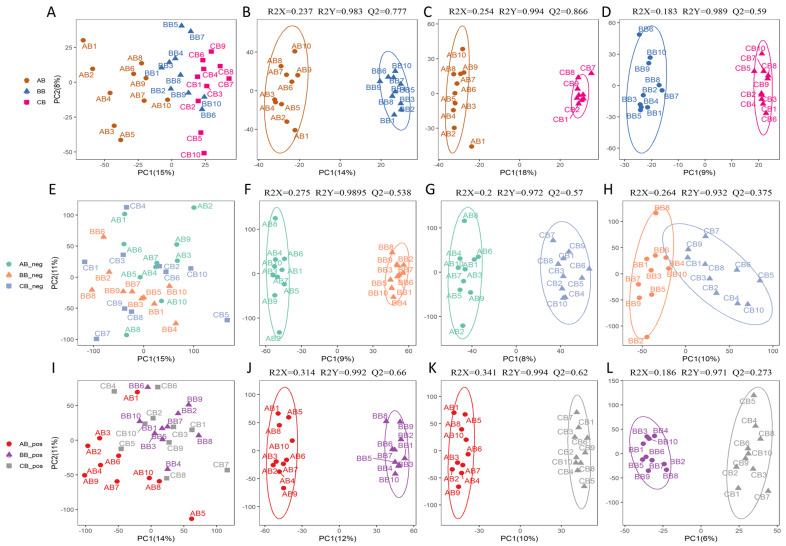
Multivariate statistical analysis of metabolites in breast meat of Beijing-You chicken at 90, 120, and 150 d. (**A**–**D**) Metabolites detected via GC-MS; (**E**–**H**) metabolites detected via LC-MS in negative mode; (**I**–**L**) metabolites detected via LC-MS in positive mode. (**A**,**E**,**I**) PCA plot based on all metabolites; (**B**–**D**,**F**–**H**,**J**–**L**) OPLS-DA plot based on all metabolites; AB, BB, and CB: breast meat samples from Beijing-You chicken at 90, 120, and 150 d, respectively.

To explore the differences in metabolites in breast meat at different ages, we integrated GC-MS and LC-MS to detect the metabolites in breast meat of BYC at 90, 120, and 150 d (ten samples for each age). PCA and OPLS-DA plots based on the GC-MS metabolite profile showed that the samples were primarily divided into three clusters according to chronological age: 90, 120, and 150 d (Figure 1A–D). Moreover, the OPLS-DA models all exhibited high accuracy within model interpretation (R2Y ≥ 0.983) and prediction capability (Q2 ≥ 0.590), indicating that they were reliable for explaining metabolite differences. In addition, both positive and negative modes were carried out in LC-MS to detect metabolites. The PCA plots illustrated that the samples from 90 d and 120 d could be distinguished clearly. For the samples from 150 d, they were mixed with the samples from 120 d (Figure 1E,I). In the unsupervised model of OPLS-DA, the samples could be distinguished by different ages completely (Figure 1F–H,J–L). The comparison groups of 120 d vs. 90 d and 150 d vs. 90 d showed high interpretation capability (R2Y ≥ 0.972) as well as high prediction capability (Q2 ≥ 0.538). For the group of 150 d vs. 120 d, it exhibited high interpretation capability (R2Y ≥ 0.932) but weak prediction capability (Q2 = 0.375 and 0.273, respectively). 

#### 3.3.2. Differential Metabolites’ Analysis

**Figure 2:**
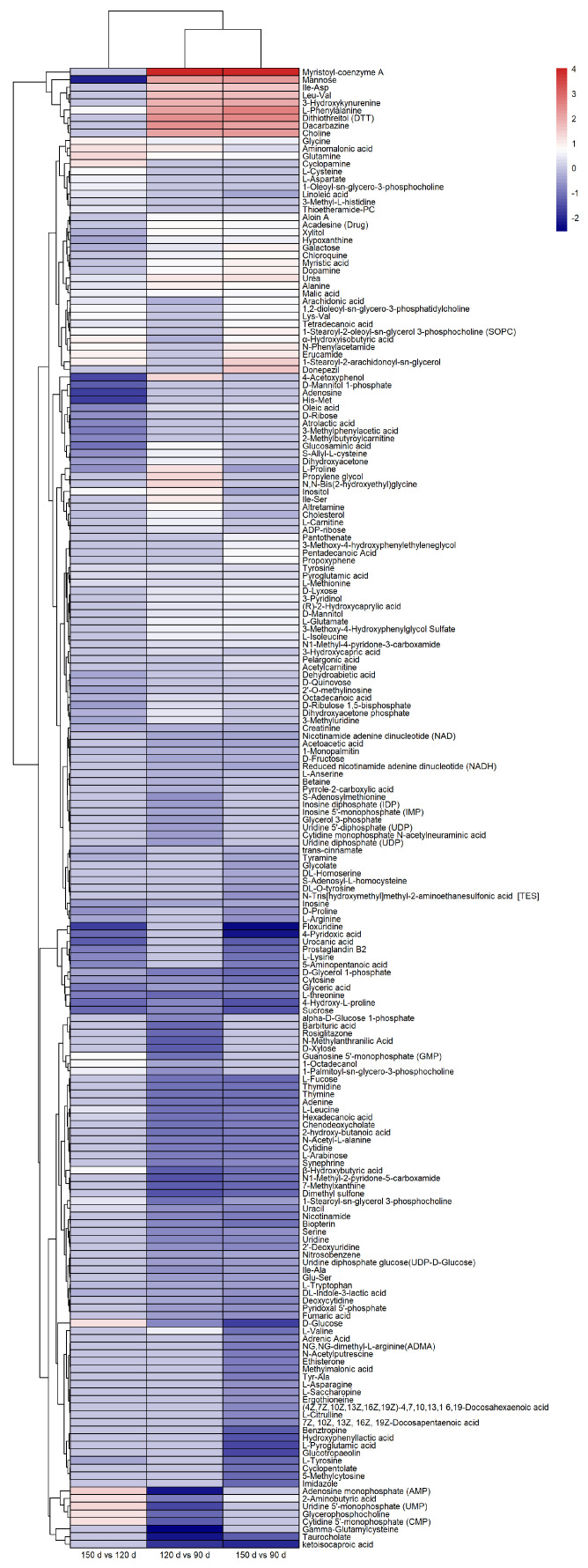
Heatmap of different metabolites at different ages.

**Figure 3:**
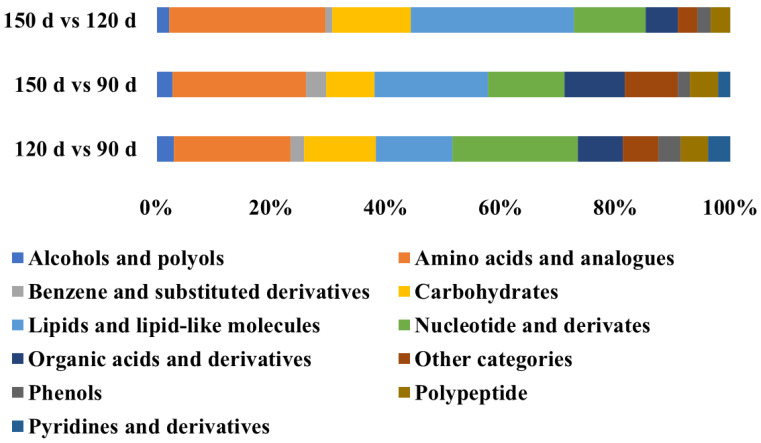
Categorization of differential metabolites.

The differential metabolites were identified based on the criteria of VIP scores (VIP > 1) as well as the *t*-test (*p* < 0.05). For LC-MS, a total of 171 and 166 compounds that differed between the age groups were identified based on the positive mode and negative mode, respectively. The metabolites from the positive and negative mode were integrated for a concise presentation in the following analysis. In the comparison group of 120 d vs. 90 d, 42 and 90 metabolites with significant differences were screened in GC-MS and LC-MS, including 4 metabolites detected using both approaches (Table 4 and Table S3, Figure 2). That is, there were 128 differential metabolites between 120 d and 90 d. For the group of 150 d vs. 90 d, there were 46 and 103 differential metabolites in GC-MS and LC-MS, with 7 overlapped. In addition, 88 unique metabolites and 1 overlapped metabolite were found to differ between 150 d and 120 d, including 40 from GC-MS and 49 from LC-MS. Additionally, the categorization of differential metabolites was performed. Those metabolites were mainly summarized into 11 categories, of which amino acids and their analogs, lipids, and lipid-like molecules were the major ones (Figure 3).

**Figure 4:**
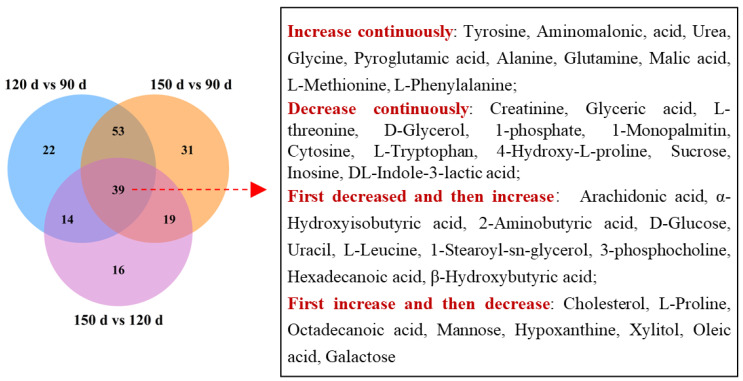
Venn diagram of differential metabolites.

The metabolites found in all three comparison groups were affected by age continuously, which was worthy of our further attention. There were 39 common metabolites present in all comparison groups (Figure 4). The concentration trend of those overlapped metabolites was analyzed according to fold changes. It was found that 10 metabolites (tyrosine, aminomalonic acid, urea, glycine, pyroglutamic acid, alanine, glutamine, malic acid, methionine, phenylalanine) increased with age. Meanwhile, 11 metabolites (creatinine, glyceric acid, threonine, d-glycerol 1-phosphate, 1-monopalmitin, cytosine, tryptophan, 4-hydroxy-l-proline, sucrose, inosine, dl-indole-3-lactic acid) decreased with age. In addition, arachidonic acid, α-hydroxyisobutyric acid, 2-aminobutyric acid, uracil, leucine, 1-stearoyl-*sn*-glycerol 3-phosphocholine, hexadecanoic acid, and β-hydroxybutyric acid showed the lowest concentration at 120 d. On the contrary, cholesterol, l-proline, octadecanoic acid, mannose, hypoxanthine, xylitol, oleic acid, and galactose showed the highest content at 120 d.

**Figure 5:**
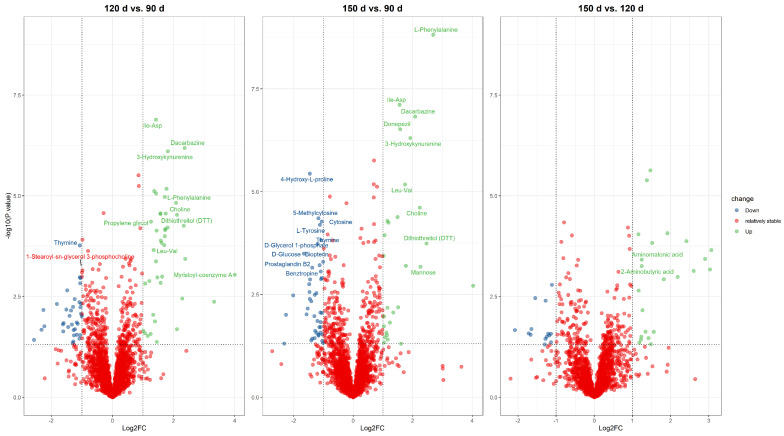
Volcano diagram of differential metabolites. The labeled dots indicate that the metabolite meets the criteria of *p* value < 0.001, VIP > 1, and |log2FC| > 1 in that comparison group.

The key differential metabolites that may influence the flavor formation of BYC were further screened according to their functions and concentrations (Figure 5). The content of sweet-tasting amino acids, such as alanine, glycine, and glutamine, continuously increased with age, and the bitter-tasting amino acids (l-tryptophan) continuously decreased with age. Umami is mainly produced by glutamate and can be enhanced by IMP, GMP, AMP, and inosine [16]. The concentration of glutamate at 90 d was significantly lower than that at 120 d and 150 d. Meanwhile, inosine had the highest content at 90 d as it was continuously down-regulated from 90 d to 150 d. For IMP, GMP, and AMP, they were significantly down-regulated at 120 d compared to 90 d and 150 d. In addition, the sweet substances, such as glucose, mannose, galactose, and xylitol, had the highest concentrations at 120 d. On the other hand, breast meat had the highest contents of oleic acid and octadecanoic acid at 120 d and arachidonic acid at 150 d. d-glycerol 1-phosphate, glyceric acid, and 1-monopalmitin had the lowest contents at 150 d as they were continuously down-regulated from 90 d to 150 d.

#### 3.3.3. Key Metabolic Pathway Analysis 

**Figure 6:**
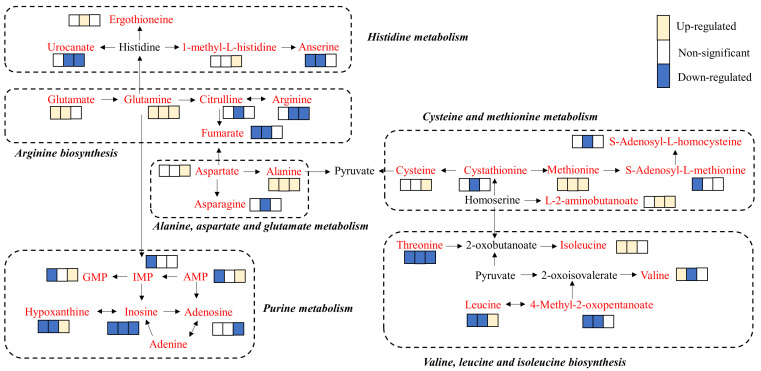
Metabolic pathways associated with flavor formation. The metabolites in red font indicate the metabolites with significant differences in this study. The three boxes next to metabolites indicate the up- and down-regulation of the metabolites in the comparison groups 120 d vs. 90 d, 150 d vs. 90 d, and 150 d vs. 120 d, respectively.

There were 20 pathways significantly enriched in breast meat, of which 6 pathways were related to flavor formation: arginine biosynthesis, valine, leucine, and isoleucine biosynthesis, histidine metabolism, alanine, aspartate, and glutamate metabolism, purine metabolism, and cysteine and methionine metabolism (Figure 6).

## 4. Discussion

The physical properties, free amino acids, and metabolites in BYC breast meat at the ages of 90, 120, and 150 days were compared. The physical properties, mainly consisting of WHC, shear force, and IMF, impact the mouthfeel directly. Water-holding capacity greatly affects meat juiciness [3]. A strong water-holding capacity results in better juiciness. The water-holding capacity is generally determined by drip loss and cooking loss. The meat tenderness is mainly reflected by shear force [17]. IMF content influences the meat palatability as well as flavor, which is an important parameter for the evaluation of meat quality [18]. Based on our results, it is shown BYC breast meat at 120 d exhibited the lowest drip loss and cooking loss, indicating the best juiciness at 120 d. The shear force increased from 90 to 150 d as well, indicating the best tenderness at 90 d. This finding is consistent with Deng et al. (2022) [19]. The shear force and IMF had no significant difference between 120 d and 150 d. Both shear force and IMF were significantly affected by the density and thickness of muscle fibers [20]. Thus, it was deduced that muscle fibers may experience significant enhancement during the period of 90 to 120 d. 

The free amino acids in breast meat were analyzed as they contribute a lot to meat flavor and human health. Firstly, free amino acids are not only important for nutrition but also the basis of protein synthesis [21]. Threonine, valine, methionine, isoleucine, leucine, phenylalanine, tryptophan, and lysine are the essential amino acids, which cannot be synthesized de novo or insufficiently relative to the body demand [22]. The contents of total free amino acids and essential amino acids decreased significantly from 90 to 120 d but had no significant difference between 120 and 150 d. This implies that the deposition of free amino acids in breast meat gradually leveled off from 120 d. In addition, free amino acids are water-soluble substances and have an important effect on the taste of meat. Glutamic acid and aspartic acid are the main contributors to umami [23]. Alanine, serine, threonine, glycine, lysine, proline, glutamine, and hydroxyproline have a sweet taste, while histidine, arginine, methionine, valine, tryptophan, tyrosine, isoleucine, leucine, and phenylalanine have a bitter taste [5]. In this study, the bitter amino acid content showed a significant decrease from 90 to 120 d. In addition, methionine, a sulfur-containing amino acid, plays an important role in meat-like aroma [24]. The content of methionine at 150 d was significantly higher than that at 90 and 120 d, suggesting prolonging the feeding age may promote the formation of meat-like aroma. 

The metabolites in meat are the main sources of flavor precursors, which play an important role in flavor development. To investigate the influence of age on metabolites, both GC-MS and LC-MS were applied to detect the metabolites in breast meat at the ages of 90, 120, and 150 days. PCA plots revealed that the metabolites at 90 d were significantly different from those at 120 and 150 d, indicating the metabolites in breast meat at 90 d were significantly different from those at 120 and 150 d. A total of 128, 142, and 88 differential metabolites were screened in the comparison groups of 120 d vs. 90 d, 150 d vs. 90 d, and 150 d vs. 120 d. The metabolite classification found that amino acids and lipids were the main differential metabolites, indicating the expression of amino acids and lipids was significantly affected by age. What’s more, the key pathways and differential metabolites associated with flavor and nutrition were identified. It was shown the pathways of arginine biosynthesis, valine, leucine, and isoleucine biosynthesis, histidine metabolism, alanine, aspartate, and glutamate metabolism, purine metabolism, and cysteine and methionine metabolism were involved in the formation of flavor during BYC development. Of note, Ge et al. (2023) also found that arginine biosynthesis, purine metabolism, and alanine, aspartic acid, and glutamic acid metabolism were the main metabolic pathways that affected breast meat flavor of BYC [25].

Methionine, cysteine, and cystathionine were enriched in the pathway of cysteine and methionine metabolism. Methionine, cysteine, and cystathionine are sulfur-containing substances, which play an important role in meat-like aroma [26]. In this study, the concentrations of methionine, cysteine, and cystathionine increased with age. Anserine was enriched in the pathway of histidine metabolism. It has been reported that anserine and ergothioneine have important physiological roles in antioxidative as well as anti-inflammatory reactions [27,28,29]. The content of anserine at 90 d was significantly higher than that at 120 and 150 d, suggesting age has negative effect on the deposition of anserine. IMP, GMP, AMP, and inosine were enriched in the pathway of purine metabolism. Glutamate, glutamine, and arginine were enriched in the pathway of arginine biosynthesis. Glutamate, IMP, GMP, AMP, and inosine play important roles in umami [30]. Glutamate had the highest content at 90 d. The content of inosine increased from 90 to 150 d. Meanwhile, IMP, GMP, and AMP had the lowest concentrations at 120 d. Since those umami substances showed different trends with age, further experiments are needed to explore the impact of age on umami. In addition, alanine was enriched in the pathway of alanine, aspartic acid, and glutamate metabolism. Arginine has an effect on the meat color and shear force of broiler meat [31]. Arginine had the highest concentration at 150 days of age. Lipids are degraded to produce fatty acids during cooking and then oxidized to generate hydroperoxides [7]. Finally, the hydroperoxides further broke down into odor-active volatile, such as aldehydes, alcohols, and ketones, which contributed to the odor of food. It was reported that phospholipids had a greater impact on meat flavor than triacylglycerols. Additionally, lipids can also act as solvents for volatile compounds produced in thermal reactions [32]. In this study, glycerol 1-phosphate, 1-monopalmitin, and hexadecanoic acid had the highest contents at 90 days. Octadecanoic acid and oleic acid have the highest contents at 120 days. Arachidonic acid has the highest content at 150 d. These results showed that the dominant expressed lipids varied with age. However, how the above lipid compounds affect the meat flavor still remains unclear; experiments are needed to investigate the specific contribution to flavor formation for those lipids above in the future. 

## 5. Conclusions

The physicochemical properties, free amino acids, and metabolites of Beijing-You chicken breast meat were investigated. The results showed that shear force and IMF content significantly increased from 90 to 120 days of age. The contents of total free amino acids and essential amino acids at the age of 90 days were significantly higher than those at the ages of 120 and 150 days. The contents of umami amino acid, bitter amino acid, and sweet amino acid had no significant differences among 90, 120, and 150 days of age. In addition, both GC-MS and LC-MS were performed to detect the metabolites in breast meat. A total of 128, 142, and 88 differential metabolites were identified in the comparison groups of 120 d vs. 90 d, 150 d vs. 90 d, and 150 d vs. 120 d, of which amino acids and lipids were the main differential metabolites. What’s more, arginine biosynthesis, valine, leucine, and isoleucine biosynthesis, histidine metabolism, purine metabolism, and cysteine and methionine metabolism were the main pathways that may have been involved in the formation of flavor during BYC development. Meanwhile, we found that some metabolites associated with flavor formation, such as methionine, cysteine, glutamate, glucose, arachidonic acid, glycerol 1-phosphate, and anserine, were significantly affected by age. Overall, our results showed that the meat quality and metabolites at 90 days of age were significantly different from those at the ages of 120 and 150 days, which reveals that attention should be focused on the period of 90 to 120 days to track the flavor formation in the future. 

## Figures and Tables

**Table 1:** Physical properties of Beijing -You chicken breast meat aged for 90, 120, and 150 days.

Terms	90 d	120 d	150 d
Drip loss (%)	3.85 ± 1.02 ^a^	2.83 ± 0.78 ^b^	3.61 ± 1.11 ^a^
Cooking loss (%)	21.65 ± 2.49 ^a^	14.40 ± 3.29 ^c^	18.41 ± 2.26 ^b^
Shear force (N)	19.21 ± 5.31 ^b^	29.15 ± 8.46 ^a^	30.62 ± 6.90 ^a^
Intramuscular fat (%)	2.42 ± 2.06 ^b^	4.68 ± 1.97 ^a^	5.66 ± 2.42 ^a^
Dry matter (%)	27.03 ± 0.83 ^a^	26.84 ± 0.75 ^ab^	26.42 ± 0.64 ^b^

^a, b, c^ Different letters within the same row indicate significant differences between different ages at *p* < 0.05 (*n* = 15). The results are shown as ‘mean ± SD’.

**Table 2:** The contents of free amino acids in breast meat of Beijing-You chicken at 90, 120, and 150 days of age.

Free Amino Acids (mg/g)	90 d	120 d	150 d
Aspartic acid	0.041 ± 0.009	0.041 ± 0.015	0.037 ± 0.022
Threonine	0.313 ± 0.100	0.315 ± 0.163	0.298 ± 0.115
Serine	0.235 ± 0.040 ^a^	0.165 ± 0.049 ^b^	0.210 ± 0.045 ^a^
Glutamic acid	0.315 ± 0.075	0.336 ± 0.148	0.351 ± 0.123
Glycine	0.213 ± 0.033 ^a^	0.162 ± 0.034 ^b^	0.148 ± 0.027 ^b^
Alanine	0.309 ± 0.093 ^a^	0.312 ± 0.086 ^a^	0.279 ± 0.102 ^b^
Citrulline	ND	ND	ND
Valine	0.145 ± 0.028 ^a^	0.078 ± 0.027 ^b^	0.088 ± 0.025 ^b^
Cystine	ND	ND	ND
Methionine	0.026 ± 0.010 ^b^	0.026 ± 0.012 ^b^	0.042 ± 0.011 ^a^
Isoleucine	0.088 ± 0.018 ^a^	0.044 ± 0.020 ^b^	0.053 ± 0.016 ^b^
Leucine	0.176 ± 0.030 ^a^	0.098 ± 0.040 ^b^	0.114 ± 0.032 ^b^
Tyrosine	0.099 ± 0.024 ^a^	0.070 ± 0.025 ^b^	0.062 ± 0.026 ^b^
Phenylalanine	0.071 ± 0.019	0.067 ± 0.022	0.061 ± 0.027
Tryptophan	ND	ND	ND
Lysine	0.210 ± 0.038 ^a^	0.144 ± 0.038 ^b^	0.120 ± 0.035 ^b^
Histidine	0.062 ± 0.021 ^a^	0.029 ± 0.033 ^b^	0.047 ± 0.028 ^ab^
Arginine	0.160 ± 0.039 ^a^	0.112 ± 0.038 ^b^	0.087 ± 0.04 ^b^
Proline	ND	ND	0.009 ± 0.02

ND, the amino acid was not detected or the content of this amino acid was less than 0.0001 mg/g; ^a, b^ different letters within the same row indicate significant differences between different ages at *p* < 0.05 (*n* = 15). The results are shown as ‘mean ± SD’.

**Table 3:** The contents of different types of free amino acids.

Terms	90 d	120 d	150 d
TAA (mg/g)	2.46 ± 0.38 ^a^	1.99 ± 0.57 ^b^	2.01 ± 0.42 ^b^
EAA (mg/g)	1.03 ± 0.18 ^a^	0.76 ± 0.28 ^b^	0.78 ± 0.2 ^b^
UAA (mg/g)	0.36 ± 0.07	0.38 ± 0.16	0.39 ± 0.12
SAA (mg/g)	1.07 ± 0.18	0.95 ± 0.27	0.94 ± 0.21
BAA (mg/g)	0.74 ± 0.13 ^a^	0.47 ± 0.15 ^b^	0.46 ± 0.13 ^b^
EAA/TAA	41.82	37.73	38.35
UAA/TAA	16.7	17.68	18.21
SAA/TAA	50.25	44.76	44.32
BAA/TAA	34.67	21.98	21.81

UAA: umami amino acid; SAA, sweet amino acid; BAA, bitter amino acid; EAA: essential amino acid; TAA: total free amino acid; ^a, b^ different letters within the same row indicate significant differences between different ages at *p* < 0.05 (*n* = 15).

**Table 4:** Statistics of differential metabolites between different ages.

Terms	120 d vs. 90 d	150 d vs. 90 d	150 d vs. 120 d
GC-MS	42	46	40
LC-MS	90	103	49
^1^ Overlapped	4	7	1
^2^ Unique	128	142	88

^1^ Overlapped metabolites in GC-MS and LC-MS; ^2^ unique metabolites in GC-MS and LC-MS.

## Data Availability

The data relevant to this study are available from the corresponding author upon reasonable request.

## Supplementary Materials

The following supporting information can be downloaded at: https://www.mdpi.com/article/10.3390/ani13213419/s1, Table S1: Sample information used in this study; Table S2: The content of free amino acids measured by amino acid analyzer. Table S3: The significantly differential metabolites in breast meat at different growth periods of Beijing-You chicken.
